# Highly sensitive multiplexed colorimetric lateral flow immunoassay by plasmon-controlled metal–silica isoform nanocomposites: PINs

**DOI:** 10.1186/s40580-024-00449-y

**Published:** 2024-10-24

**Authors:** Minsup Shin, Wooyeon Kim, Kwanghee Yoo, Hye-Seong Cho, Sohyeon Jang, Han-Joo Bae, Jaehyun An, Jong-chan Lee, Hyejin Chang, Dong-Eun Kim, Jaehi Kim, Luke P. Lee, Bong-Hyun Jun

**Affiliations:** 1 Department of Bioscience and Biotechnology, Konkuk University, Seoul, 05029 Republic of Korea; 2 Company of BioSquare, Hwaseong, 18449 Republic of Korea; 3 Department of Internal Medicine, Seoul National University Bundang Hospital, Seongnam, 13620 Republic of Korea; 4 Division of Science Education, Kangwon National University, Chuncheon, 24341 Republic of Korea; 5 Renal Division and Division of Engineering in Medicine, Department of Medicine, Brigham and Women’s Hospital, Harvard Medical School, Boston, MA 02115 USA; 6 Department of Bioengineering, Department of Electrical Engineering and Computer Science, University of California at Berkeley, Berkeley, CA 94720 USA; 7 Institute of Quantum Biophysics, Department of Biophysics, Sungkyunkwan University, Suwon, 16419 Republic of Korea

**Keywords:** Colorimetric lateral flow immunoassays, Multicolored metal nanoparticles, Silica nanoparticles, Seed mediated growth method, Multiplex analysis

## Abstract

Lateral flow assay (LFA) systems use metal nanoparticles for rapid and convenient target detection and are extensively studied for the diagnostics of various diseases. Gold nanoparticles (AuNPs) are often used as probes in LFAs, displaying a single red color. However, there is a high demand for colorimetric LFAs to detect multiple biomarkers, requiring the use of multicolored NPs. Here, we present a highly sensitive multiplexed colorimetric lateral flow immunoassay by multicolored Plasmon-controlled metal–silica Isoform Nanocomposites (PINs). We utilized the localized surface plasmon resonance effect to create multi-colored PINs by precisely adjusting the distance between the NPs on the surface of PINs through the controlled addition of reduced gold and silver precursors. Through simulations, we also confirmed that the distance between nanoparticles on the surface of PINs significantly affects the color and colorimetric signal intensity of the PINs. We achieved multicolored PINs that exhibit stronger colorimetric signals, offering a new solution for LFA detection with high sensitivity and a 33 times reduced limit of detection (LOD) while maintaining consistent size deviations within 5%. We expect that our PINs-based colorimetric LFA will facilitate the sensitive and simultaneous detection of multiple biomarkers in point-of-care testing.

**Graphical Abstract:**

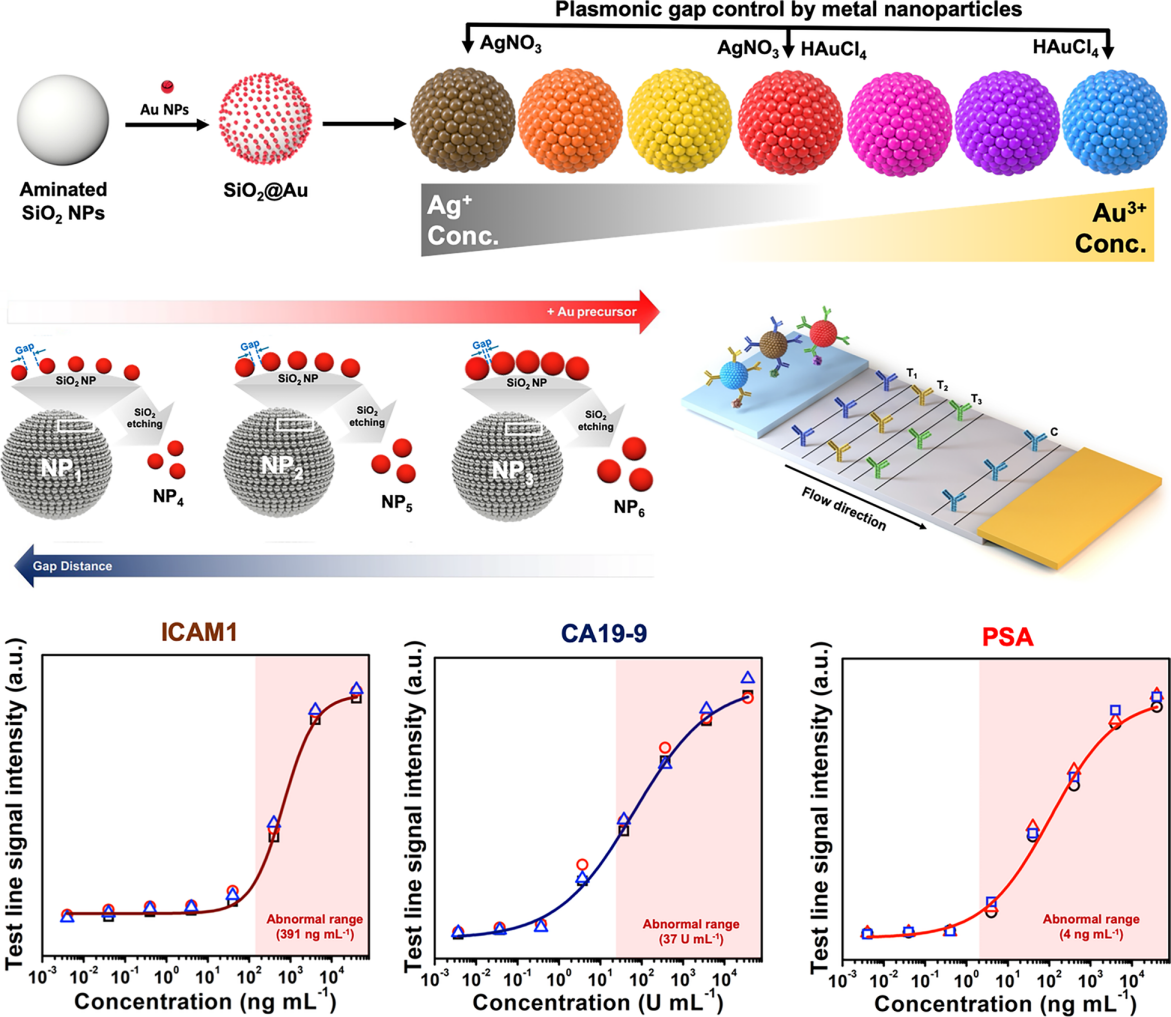

**Supplementary Information:**

The online version contains supplementary material available at 10.1186/s40580-024-00449-y.

## Introduction

The point-of-care test (POCT) is essential for diagnosing several critical diseases [1, 2]. Unlike traditional diagnostic methods, the POCT does not require complex processes involving expensive instruments or specialized operators. This advantage enables people to conduct frequent self-diagnosis at home, facilitating early detection and diagnosis of diseases. Early diagnosis is crucial for preventing fatalities from conditions like pancreatic cancer, which are challenging to treat in advanced stages [3].

Various diagnostic methods, such as lateral flow assays [4–7], electrochemical sensors [8, 9], and microfluidic sensors [10, 11], have been developed and applied in POCTs. Colorimetric lateral flow assays (CLFAs) are commonly used owing to their simplicity, ease of use, and cost-effectiveness. CLFAs enable the visual detection of target biomarkers unless their concentration in the sample is significantly lower than the limit of detection (LOD) [12, 13].

Metallic nanoparticles (NPs), especially gold (Au) NPs [14–17], are commonly used as colorimetric probes for CLFAs owing to their unique physical properties. Spherical AuNPs exhibit an intense red color that is visible to the naked eye. The color of AuNPs can vary based on the size and morphology of the NPs, which is determined by their fabrication methods [18–23]. Moreover, AuNPs have a strong affinity for thiol [24, 25] or amine [26] groups; therefore, various kinds of desired ligands can be easily attached to the surface of NPs if the ligands have corresponding functional groups. This high stability provides signal repeatability to the AuNPs probes, even when stored for a long time. Based on these characteristics, AuNPs are widely used in CLFAs to detect various biomarkers and diagnose diseases [15, 27, 28]. Recently, heterogeneous metallic NPs, such as core–shell type NPs, have also been employed as CLFA probes [4, 5, 29–32]. These heterogeneous metallic NPs exhibit stronger colorimetric signals compared with homogeneous metallic NPs. Additionally, metallic NP-assembled silica NPs have been used as nanoprobes in lateral flow immunoassays (LFIAs) [12, 13]. These fabricated nanoprobes exhibit stronger colorimetric signals than traditional AuNPs, facilitating sensitive biomarker detection.

Despite these advantages, multiplex analyses using CLFAs with metallic NPs are limited. In previous studies, NPs of the same color were often used as probes for different targets [33–35], making it impossible to distinguish the specific probe bound to the test line for detecting a particular target. As a result, the presence of nonspecific binding was uncertain. Therefore, various probes with multiple colors are essential for multiplex analysis via CLFAs. Although the color of NPs can be adjusted by altering their size and/or morphology, this approach can affect other properties of NPs, such as surface area and antibody attachment [36, 37]. This affects the LOD, one of the most essential values for sensing systems, even though the target biomarker is the same when NPs of different colors are used. This problem is a critical obstacle for multiplex analysis using CLFAs; therefore, developing novel colorimetric probes with multiple colors and without dramatic differences in size and morphology is essential for multiplex analysis using CLFAs.

**Fig. 1:**
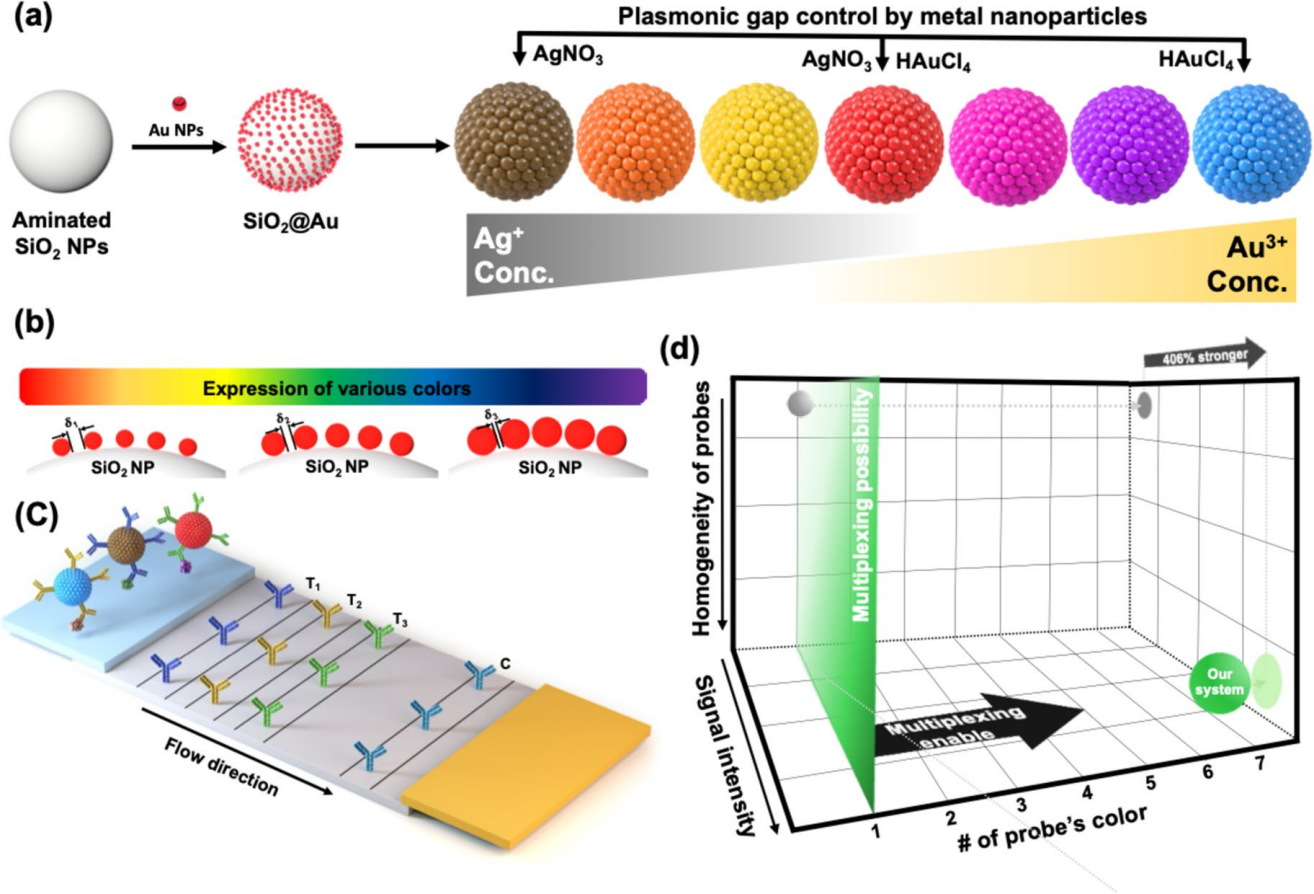
Fabrication of multicolored PINs and their application to colorimetric lateral flow immunoassays (CLFA). **a** Synthetic scheme of PINs via seed-mediated growth method. By controlling the composition and amount of used metal precursors, 7 kinds of PINs were fabricated. **b** Correlation between nanogaps and size of metal nanoparticles on silica nanoparticle (SiO_2_ NP). The gap distance between each metal nanoparticle was closer as the size of metal nanoparticles was increased, and as a result, the color of PINs varied. **c** Schematic illustration of CLFA with fabricated PINs for analysis of multiple targets. PINs with different antibodies were prepared to detect the corresponding targets. Antibodies for capturing each target were dispensed onto T_1_, T_2_, and T_3_ lines, respectively. Immunoglobulin G was dispensed onto the C line as a control for capturing antibody-conjugated PINs. **d** A graph showing the comparison of our multiplex CLFA system and existing CLFA. Based on PIN probes, which have various colors and show more intense colorimetric signals than existing probes for CLFA, multiple targets can be detected simultaneously, and the intensity of colorimetric signals was 406% enhanced with our CLFA system

In this study, we report a highly sensitive multiplexed colorimetric lateral flow immunoassay by multicolored Plasmon-controlled metal–silica Isoform Nanocomposites (PINs). We applied the localized surface plasmon resonance effect to create multi-colored PINs by precisely adjusting the distance between the NPs on the surface of PINs through the controlled addition of reduced gold and silver precursors. We accomplished seven types of PINs, representing navy, purple, magenta, red, yellow, orange, and brown, were fabricated by attaching Au nanoseeds to amine-functionalized SiO_2_ NPs and then growing metal NPs around the seeds via a seed-mediated growth method (Fig. 1a). The size of the metal NPs and the gap distance between them, which affect the enhancement of the electric field and UV–Vis extinction spectra, were controlled by adjusting the amount of metal precursors (Fig. 1b). After surface modification, the fabricated PINs were conjugated with antibodies and used in a CLFA system to confirm their performance as colorimetric probes (Fig. 1c). Unlike traditional systems, our CLFA utilized distinctly colored probes, clearly distinguishing between different targets. The PINs, having a similar structure with densely packed metal NPs on silica, showed similar sensitivities and a colorimetric signal intensity 406% higher than AuNPs typically used in CLFA systems (Fig. 1d). This CLFA system offers sensitive detection and the capability for multiplex analysis, making it a powerful tool for diagnosing critical or highly pathogenic diseases, such as cancers or avian influenza.

## Results and discussion

### Design and simulation of multi-colored PINs

**Fig. 2:**
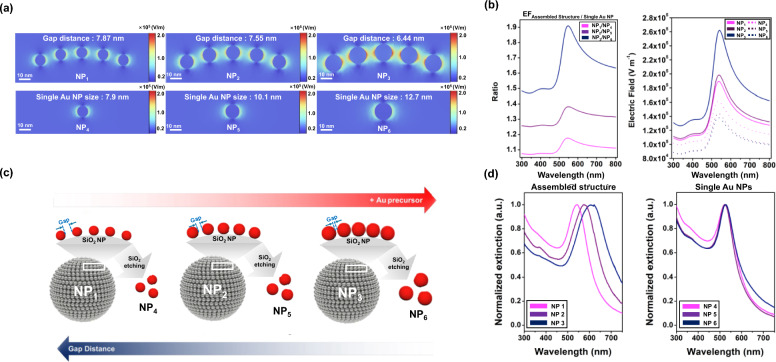
**a** Electric field distributions of five AuNPs (NP_1_, NP_2_, and NP_3_) and single AuNP (NP_4_, NP_5_, and NP_6_). The size of single AuNP was increased from NP_1_ to NP_3_, and it in NP_1_, NP_2_, and NP_3_ was the same with NP_4_, NP_5_, and NP_6_, respectively. The electric fields were simulated with COMSOL Multiphysics. **b** Spectra of maximum electric field intensity in each NP (left) and the ratio between assembled structure and single AuNP (right; NP_1_/NP_4_, NP_2_/NP_5_, and NP_3_/NP_6_). **c** Schematic images of NP_1_ to NP_6_. NP_4_ was fabricated etching of silica from NP_1,_ in which AuNPs with the smallest size were assembled onto the surface of SiO_2_, and NP_5_ and NP_6_ were fabricated from NP_2_ and NP_3_ in the same manner, respectively. **d** UV–Vis extinction spectra of NP_1_ to NP_3_ (left) and NP_4_ to NP_6_ (right)

Prior to fabricating the NPs, we conducted a simulation to reveal the effects of the size and gap distance of the metal NPs on the localized surface plasmon resonance and electric field around the NPs. In the seed-mediated growth system, two key characteristics were observed: (1) the size of the AuNPs increased as the amount of gold precursor used for fabrication increased; (2) the gap distance between the AuNPs decreased as the particles grew around a limited number of Au nanoseeds on silica nanotemplates. Based on these two characteristics, the electric field's intensity changes which according to the size of the AuNPs and the gap distance between the AuNPs were simulated. We modeled six kinds of simplified virtual nanostructure (NP_1_–NP_6_) for the simulation. NP_1_ to NP_3_ consisted of five AuNPs with distinct sizes and gap distances, with NP_1_ having the smallest and widest gaps and NP_3_ having the largest and closest gaps. NP_4_, NP_5_, and NP_6_ were single AuNPs with the same size as NP_1_, NP_2_, and NP_3_, respectively. The simulation predicted that nanogaps between the AuNPs would enhance the electric fields (Fig. 2a). As the size of the AuNPs increased and the gap distance between the AuNPs decreased, the intensity of the electric field increased (NP_3_ > NP_2_ > NP_1_). Conversely, for single AuNPs, the intensity of the electric field slightly increased as the size of the AuNPs decreased (NP_4_ > NP_5_ > NP_6_). Figure 2b shows the UV–Vis spectra of the electric field intensity. The wavelength that produced an electric field with maximum intensity was approximately 540 nm for all particles. The electric field intensity increased as the size of the single AuNPs decreased and significantly increased as the gap distance between the assembled AuNPs decreased. Moreover, the ratio of electric field intensity between assembled structures and single AuNPs was the highest when the gap distance was the closest (1.91), decreasing to 1.38 and 1.18 as the gap distance increased.

To determine the relationship between the simulation and experimental results, we fabricated SiO_2_@Au NPs by growing AuNPs around Au nanoseeds on silica NPs using a seed-mediated method (Fig. 2c). First, we created three types of NPs (NP_1_, NP_2_, and NP_3_) by varying the amounts of gold precursor to control the size of the AuNPs and the gap distance between each AuNP. Subsequently, the silica nanotemplates of each NP were etched to obtain single AuNPs (NP_4_, NP_5_, and NP_6_). The UV–Vis extinction spectra of each type of NP were analyzed to reveal the relationship between the electric field enhancement and the UV–Vis extinction spectra (Fig. 2d). NP_1_, NP_2_, and NP_3_, which featured assembled AuNPs with nanogaps, showed similar UV–Vis extinction spectra patterns; however, the maximum extinction wavelength shifted toward longer wavelengths as the amount of gold precursor increased (from 542 nm in NP_1_ to 622 nm in NP_3_). In comparison, NP_4_, NP_5_, and NP_6_ showed similar maximum extinction wavelengths, regardless of the size or origin of the AuNPs. These results suggested that the UV–Vis extinction spectra of each NP could be influenced by electric field enhancement. Specifically, greater electric field enhancement induced a red shift in the maximum UV–Vis extinction wavelength. This indicated that the color of the NPs could be controlled by adjusting the amount of metal precursor used, affecting the size and gap distance of the metal NPs on the silica nanotemplates.

### Characterization of multi-colored PINs

Based on the simulation results, we fabricated seven different colors of PINs with uniform particle sizes representing red, yellow, orange, brown, magenta, purple, and navy colors using the seed-mediated growth method. Aminated SiO_2_ NPs with an average size of approximately 164 nm were prepared as the core. Subsequently, Au nanoseeds with an average size of approximately 5 nm were attached to the surface of the aminated SiO_2_ NPs to fabricate SiO_2_@Au, the precursor for PINs. Silver (Ag) and/or AuNPs were grown around the Au nanoseeds to vary the color of the NPs, which acted as seeds for the growth of metallic NPs under different precursor concentration conditions. Growing AgNPs fabricated yellow, orange, and brown PINs, whereas magenta, purple, and navy PINs were fabricated by growing AuNPs. Red PINs were fabricated by growing alloyed Au and Ag NPs around Au nanoseeds.

**Fig. 3:**
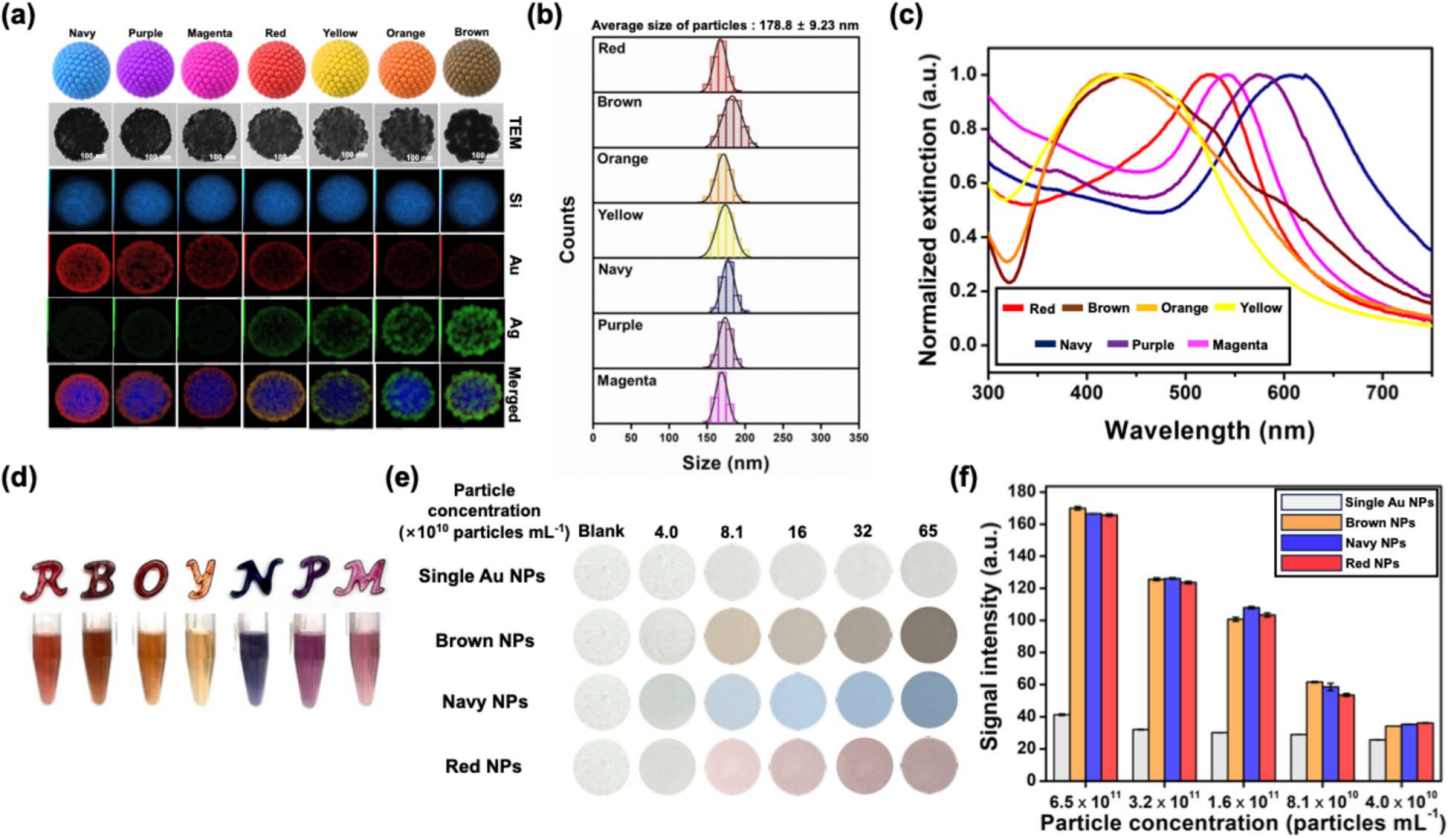
**a** Transmission electron microscopy (TEM) images and energy dispersive spectroscopy (EDS) mapping images of synthesized multi-colored PINs. **b** Size distribution graph of each PIN. **c** UV–Vis extinction spectra of each PIN. **d** Photograph image of PINs after drying as alphabet, which represented the initial color (above R from red, B from brown, O from orange, Y from yellow, N from the navy, P from purple, and M from magenta) and dispersed in water (below). **e** Photograph images of the NC membrane after dropping and drying the single AuNP with 20 nm size, brown, navy, and red PINs. **f** Colorimetric signal intensity of single AuNP with 20 nm size, brown, navy, and red PINs at the various particle concentrations

Figure 3a shows the transmission electron microscopy (TEM) images and energy-dispersive spectroscopy (EDS) results of the fabricated PINs. The average size of the metal NPs on the surface of the NPs increased as the amounts of added metal precursors (HAuCl_4_ and AgNO_3_) and ascorbic acid increased. Brown, orange, and yellow PINs, in which AgNO_3_ was added as a precursor, grew in a lump form with the loss of their original spherical morphology owing to the characteristics of Ag. In contrast, the metal NPs on the red, magenta, purple, and navy PINs, in which HAuCl_4_ was added as a precursor, retained their spherical morphologies. The average sizes of the metal NPs on the brown, orange, yellow, red, magenta, purple, and navy PINs were 18.2 nm, 11.9 nm, 7.6 nm, 5.4 nm, 7.9 nm, 10.1 nm, and 12.7 nm, respectively. The EDS results revealed the composition of the metal NPs on each PIN: Ag (yellow, orange, and brown PINs), Au (magenta, purple, and navy PINs), and Au–Ag (red PINs).

Figure 3b shows the size distribution of the PINs. The average size of the PIN was 178.8 ± 9.2 nm, indicating a uniform size and high monodispersity with a standard deviation of only 4.96% of the average size [38–40]. This uniformity is crucial for reliable multiplex analysis via LFIA. In a previously reported method for multiplex analysis using LFIA [41, 42], the morphologies and surface areas of the nanoprobes differed significantly according to their colors. Consequently, the number of attached antibodies and the mobility of the nanoprobes differed, leading to variations in the LOD for the same target. Thus, our PINs, with similar sizes and morphologies, are well-suited for enhancing LFIA reliability.

To elucidate the color variations in the PINs, we confirmed the color and measured the UV–Vis extinction spectra of each PIN (Fig. 3c). The spectra revealed characteristic extinction patterns based on the type of metal precursor added. The yellow, orange, and brown PINs with AgNPs exhibited similar maximum extinction wavelengths (430, 425, and 442 nm, respectively). According to the reported paper, AgNPs which grown on yellow, orange, and brown PINs assembled with gap distance of about 4 to 8 nm represented similar maximum extinction wavelength and larger maximum extinction cross section compared with gap distance with 0 to 4 nm [43]. While growing, AgNPs lost spherical shape and even merge of NPs which originally separated also could be observed, and therefore, closer gap distance which extinct the light with wavelength of 550–700 nm had appeared frequently as AgNPs were grown. Yellow, orange, and brown PINs represented different colors. As results, the degree of light extinction in the 550–700 nm range increased as the size of the AgNP increased and yellow, orange, and brown PINs represented different colors. In contrast to AgNPs, PINs with AuNPs exhibited different maximum extinction wavelengths (542 nm for magenta, 574 nm for purple, and 622 nm for navy PINs) with similar spectral patterns, because extinction spectra of AuNPs were red-shifted as the gap distance between AuNPs was being closer [44–46]. The red PINs showed maximum extinction at 524 nm, with a spectral pattern similar to that of the PINs with AuNPs, except in the 300–450 nm range. Based on these UV–Vis extinction spectra, the PINs showed distinguishable colors both in solution and after drying (Fig. 3d).

To determine whether the CLFA results using the fabricated PINs could be qualitatively analyzed with the naked eye and quantitatively analyzed using ImageJ, PINs and single AuNPs at various concentrations were applied to a nitrocellulose (NC) membrane and analyzed **(**Fig. 3e). Among the seven types of PINs, the brown (SiO_2_@Au@Ag), navy (SiO_2_@Au@Au), and red (SiO_2_@Au@AuAg) PINs, all of which had different metal NP compositions and distinguishable colors, were selected as probes for the CLFA. Prior to application, the signal intensity of these PINs was compared to that of the AuNPs (20 nm), which are commonly used as CLFA probes. As shown in Fig. 3e, each PIN distinctly displayed its color after drying on the NC membrane until a particle concentration of 4.0 × 10^10^ particles mL^−1^ was achieved. Conversely, the AuNPs did not show a notable red color even at a particle concentration of 6.5 × 10^11^ particles mL^−1^. Figure 3f shows the quantified signal intensity of each PIN and AuNPs using the ImageJ software. All three types of PINs showed a signal intensity that was more than four-fold higher than that of AuNPs at a particle concentration of 6.5 × 10^11^ particles mL^−1^. Even at a diluted particle concentration of 4.0 × 10^10^ particles mL^−1^, the PINs exhibited stronger signals. This strong signal intensity can be attributed to the higher light extinction of PINs compared to that of AuNPs under the same particle concentration conditions. Therefore, PINs could exhibit more vivid colors than AuNPs, even at low particle concentrations. This enables sensitive detection in CLFA systems using PIN probes. To confirm the stability of fabricated PINs after storage, we checked the color of PINs mixtures and morphology of PINs. PINs were stored in the 4℃ refrigerators after redispersed in the aqueous PVP solution, and the photograph images were taken (Figure S2). PINs mixtures retained their characteristic colors without significant change for not only within 7 days, but also even after 2 years. The morphology of stored PINs was confirmed by taken TEM images (Figure S3). As like the color of mixtures, morphology of PINs also did not represent significant change for 2 years. With these results, it was certain that our PINs can maintain their performance as the nanoprobes of CLFA system after long-term storage.

### Detection of prostate-specific antigen (PSA) using PIN-based CLFA

**Fig. 4:**
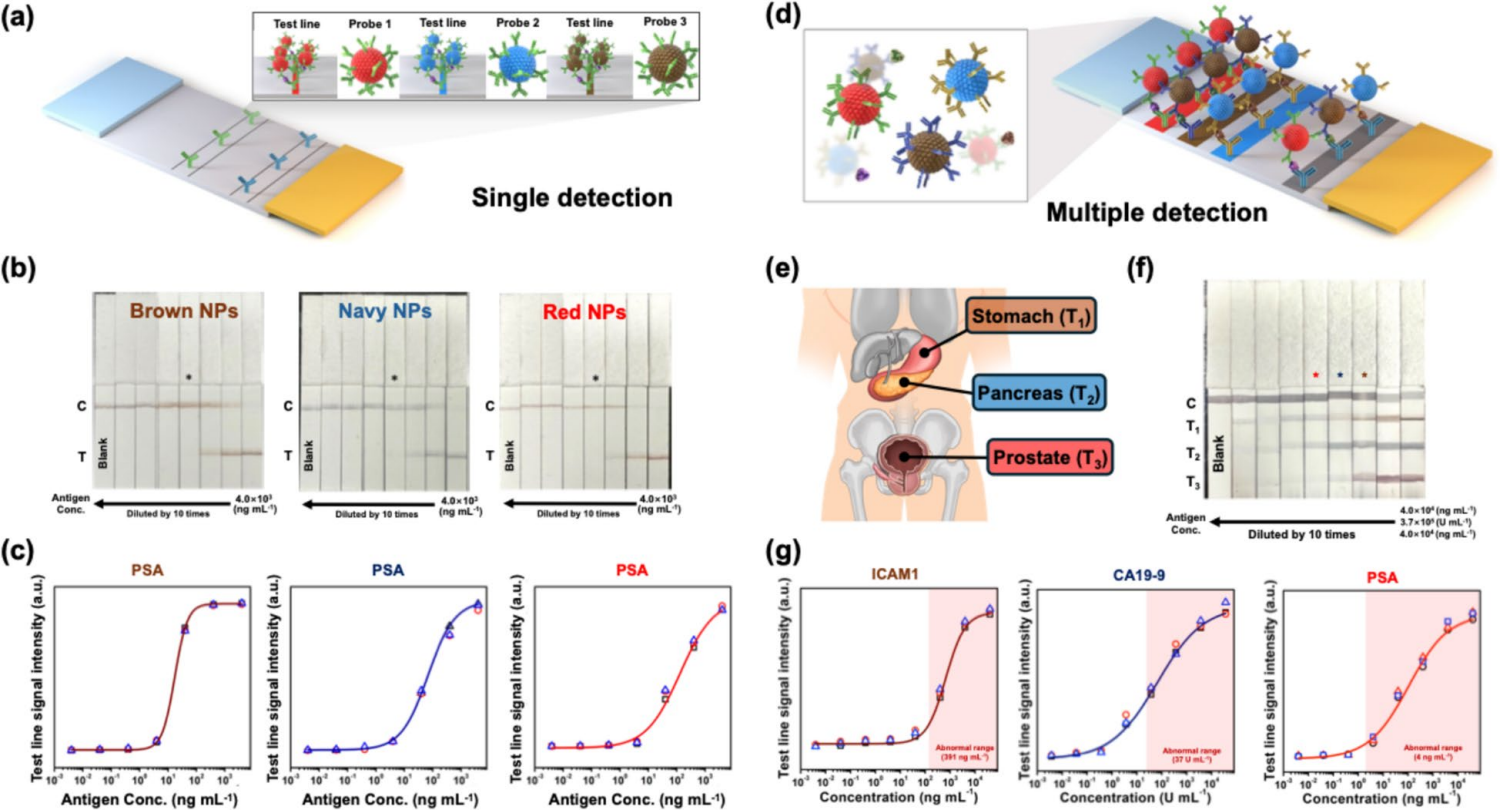
CLFA system using brown, navy, and red PINs as probes. **a** Scheme of the CLFA system for detection of prostate-specific antigen (PSA) as a single target. **b** Photograph images of CLFA strips after developing. * marks represented the concentration level with a boundary for the abnormal range of PSA (4.0 ng mL^−1^). **c** Quantitative analysis of PSA based on detection results. **d** Scheme of the CLFA system for multiplex detection of intercellular adhesion molecule 1 (ICAM1), carbohydrate antigen 19–9 (CA19-9), and PSA as multiple targets. **e** Illustration of three representative organs in which cancer can occur: Stomach (T_1_), Pancreas (T_2_), and Prostate (T_3_). ICAM1, CA19-9, and PSA are representative biomarkers for each cancer, representatively. **f** Photograph images of CLFA strips after developing. * marks represented the concentration level with a boundary for the abnormal range of each biomarker (391 ng mL^−1^ for ICAM1, 37 U mL^−1^ for CA19-9, and 4.0 ng mL^−1^ for PSA). **f** Quantitative analysis of ICAM1, CA19-9, and PSA based on detection results. The red zone represented an abnormal range of each biomarker

To evaluate the suitability of PINs as probes for CLFAs, prostate-specific antigen (PSA), a well-studied biomarker, was selected as a target (Fig. 4a) [47]. Prior to conducting the CLFA, anti-PSA capture antibodies and immunoglobulin G (IgG) were immobilized onto the test and control lines, respectively, of the NC membrane. Anti-PSA detection antibodies were conjugated with 11-mercaptoundecanoic acid (11-MUA)-introduced PINs via the EDC/sulfo-NHS reaction. PSA at various concentrations was mixed with each PIN probe and the mixture was applied to the NC membrane strip. The capture antibody-conjugated probes bound to PSA, forming complexes. When these complexes reached the test line, they were captured by the immobilized capture antibodies, making the color of the PINs visible at the test line. In contrast, probes that did not bind to PSA passed the test line and bound to IgG on the control line. PSA was detected at various concentrations using CLFAs with three types of PIN probes. Figure 4b shows photographs of the strips after PSA detection. For all three types of PIN probes, the color at the control lines became vivid and that of the test lines became fainter as the PSA concentration decreased. The color of the test lines remained visible to the naked eye until the PSA concentration was diluted to 4.0 ng mL^−1^, which is the clinical threshold for abnormal PSA levels associated with prostate cancer [48]. To determine the LOD for each PIN probe, we conducted a quantitative analysis using the ImageJ software (Fig. 4c). The LOD for brown, navy, and red PINs was 0.09 ng mL^−1^, 0.06 ng mL^−1^_,_ and 0.34 ng mL^−1^, respectively. These calculated LOD values were at least 11-fold lower than 4.0 ng mL^−1^, indicating the high sensitivity of the PIN probes. Moreover, these LOD values were significantly lower than those of another reported LFIA system which various probes such as AuNP, carbon dot, and mesoporous silica with Prussian blue, demonstrating the enhanced sensitivity of our PIN-based CLFA [49–54]. In summary, these qualitative and semi-quantitative analyses indicate that our PINs are suitable for use as probes in CLFA systems, regardless of their composition and color.

### Multiplex analysis of biomarkers using PIN-based CLFA

Based on the previous findings, we conducted a multiplex analysis of biomarkers using our PIN probes in a CLFA system. To prepare the probes, detection antibodies for intercellular adhesion molecule 1 (ICAM1), carbohydrate antigen 19-9 (CA19-9), and PSA were conjugated to brown, navy, and red PINs, respectively, after surface modification of each PIN with 11-MUA. The NC membrane was prepared by adsorbing IgG (C), ICAM1 capture antibody (T_1_), CA19-9 monoclonal antibody (T_2_), and anti-PSA capture antibody (T_3_) from the absorbent pad to the nearest side. The antigens were mixed at specific concentrations with the prepared probes, and the mixture was developed along the NC membrane strip (Fig. 4d). ICAM1, CA19-9, and PSA were selected as targets for model tests because they are key biomarkers for gastric [55], pancreatic [56], and prostate cancers, respectively (Fig. 4e).

In the multiplex analysis, each probe and antigen specifically formed a complex and bound to the corresponding capture antibodies at the designated test lines, while the unbound probes were captured by IgG on the control line. Figure 4f shows photographs of the strips after the multiplex analysis, with each test line displaying a distinct color: brown (T_1_), navy (T_2_), and red (T_3_), corresponding to ICAM1, CA19-9, and PSA, respectively. The intensity of test line colors became faint as the concentration of the biomarkers was reduced; however, the colors remained visible to the naked eye at the abnormal threshold for each biomarker (391 ng mL^−1^ for ICAM1, 37 U mL^−1^ for CA19-9, and 4.0 ng mL^−1^ for PSA). As in the model test, the LOD for each biomarker was determined through quantitative analysis using ImageJ software (Fig. 4g). The calculated LOD of ICAM1 using brown PINs was 6.65 ng mL^−1^, which is almost 60-fold lower than the 391 ng mL^−1^ threshold for gastric cancer risk [55]. The LOD for CA19-9 using navy PINs was 0.04 U mL^−1^, which is 925-fold lower than the 37 U mL^−1^ upper normal limit associated with pancreatic cancer risk [57, 58]. For PSA using red PINs, the LOD was 0.12 ng mL^−1^, which is 33-fold lower than the 4.0 ng mL^−1^ abnormal threshold, consistent with the results from the model test. In summary, ICAM1, CA19-9, and PSA were successfully detected using the newly developed multiplex CLFA system, which uses fabricated PIN probes, even at considerably lower concentrations than the abnormal range of each disease. Given these results, the developed multiplex CLFA system demonstrates the potential for simultaneous multiplex analysis of various critical diseases using minimal sample volume.

## Conclusions

We fabricated seven types of multi-colored PINs with metal NP-assembled SiO_2_ NP structures using a seed-mediated growth method with SiO_2_ NPs and Au nanoseeds. These PINs exhibited unique and distinguishable colors, with uniform average particle sizes. By varying the amounts of Ag and/or Au precursors, we controlled the morphology and composition of metal NPs on the surface of SiO_2_ NPs. Specifically, the gap distance between the metal NPs, which significantly influences their color, was adjusted. Additionally, these PINs exhibited more vivid colors than single AuNPs, which are commonly used in CLFA systems, owing to their metal NP-assembled structure. The superior optical properties of the PINs enabled the detection of PSA using a CLFA system with brown, navy, and red PINs as probes. PSA concentrations above the abnormal threshold were detectable by the naked eye. The semi-quantitative analysis determined the LOD for PSA as 0.09 ng mL^−1^, 0.06 ng mL^−1^, and 0.34 ng mL^−1^ for the brown, navy, and red PINs, respectively. These values are significantly lower than 4.0 ng mL^−1^, which is the abnormal threshold concentration of PSA. These three types of PINs were also used in a multiplex CLFA system for the detection of ICAM1, CA19-9, and PSA, which are representative biomarkers of gastric, pancreatic, and prostate cancers, respectively. Each PIN probe displayed distinct colors on the corresponding test lines, enabling the visual detection of biomarker concentrations above the abnormal range. The calculated LODs for ICAM1, CA19-9, and PSA were 6.65 ng mL^−1^, 0.04 U mL^−1^, and 0.12 ng mL^−1^, respectively, each markedly lower than the abnormal range thresholds. These findings suggest that this newly designed multiplex CLFA system will greatly improve the reliability and speed of results. It holds promise for comprehensive multiple analyses across various cancers and for detecting multiple biomarkers concurrently within a single type of cancer, potentially improving diagnostic capabilities and patient outcomes.

## Methods

### Materials and reagents

Tetraethylorthosilicate (TEOS), 3-aminopropyltriethoxysilane (APTES), gold (III) chloride trihydrate (HAuCl_4_∙3H_2_O, 99.9%), silver nitrate (AgNO_3_, 99.9%), ascorbic acid, 1-ethyl-3-(3-dimethylaminopropyl)carbodiimide hydrochloride (EDC), N-hydroxysulfosuccinimide (sulfo-NHS), sodium borohydride (NaBH_4_), 11-MUA, 4-morpholineethanesulfonic acid (MES), ethanolamine**,** 96-well plate, and poly(vinylpyrrolidone) (PVP, Mw ~ 40,000) were purchased from Sigma-Aldrich (St. Louis, MO, USA). Potassium hydroxide (KOH, 95.0%), sodium hydroxide (NaOH), hydrochloric acid (HCl, 35–37%), and ethanol (99%) were purchased from Samchun Chemical (Seoul, Republic of Korea). Ammonium hydroxide (NH_4_OH, 27%) was purchased from Daejung (Busan, Republic of Korea). Phosphate-buffered saline (PBS, pH 7.4) and 0.5% (v/v) Tween 20 in phosphate-buffered saline (PBST, pH 7.4) were purchased from DyneBio (Seongnam, Republic of Korea). The ICAM1 antibody (Ab) pair BSA and azide-free (ab288941) and recombinant human ICAM1 protein (ab82125) were purchased from Abcam (Cambridge, UK). PSA was purchased from Fitzgerald Industries (Acton, MA, USA). Goat anti-mouse IgG antibody, anti-PSA (14801) Ab, anti-PSA (14803) Ab, backing card, NC membrane, and absorbent pads were purchased from Bore Da Biotech Co., Ltd. (Seongnam, Republic of Korea). CA19-9 monoclonal antibodies (A46300 and A46400) and CA19-9 antigen (J66100) were purchased from BiosPacific Inc. (Emeryville, CA, USA). Deionized water (D.W.) was used in all experiments.

### Instruments and analyses

TEM images and EDS for elemental mapping of the NPs were obtained using a JEM-F200 (JEOL, Akishima, Tokyo, Japan) with a maximum accelerated voltage of 200 kV. The UV–Vis-NIR extinction spectra of the NPs were obtained using an Optizen UV–Vis spectrometer (Mecasys, Daejeon, Republic of Korea). The electric field distribution around the NPs was simulated using the COMSOL Multiphysics software. The solutions for the test and control lines were dispensed using an Automated Lateral Flow Reagent Dispenser (Claremont Bio, Upland, CA, USA). The colorimetric signal intensities of each line on the NC membranes were measured using the ImageJ software.

### Fabrication and amination of silica NPs (SiO_2_ NPs)

SiO_2_ NPs approximately 150 nm in size were fabricated via a modified Stöber method [59]. TEOS (1.6 mL) and NH_4_OH (3 mL) were added to absolute ethanol (40 mL) while stirring at 700 rpm with a magnetic bar. After 20 h, the SiO_2_ NPs were washed with ethanol five times via centrifugation at 8,500 rpm for 10 min. The concentration of SiO_2_ NPs was adjusted to 50 mg mL^−1^ with absolute ethanol. To introduce amine groups onto the surface of SiO_2_ NPs, 12.5 mg of SiO_2_ NPs (in 250 μL of ethanol) were mixed with APTES (15.5 μL) and NH_4_OH (10 μL). After shaking overnight, the aminated SiO_2_ NPs were washed with ethanol three times via centrifugation at 8,500 rpm for 10 min. The concentration of aminated SiO_2_ NPs was adjusted to 10 mg mL^−1^ with absolute ethanol.

### Fabrication of Au nanoseeds introduced SiO_2_ NPs (SiO_2_@Au)

Au nanoseeds (3–5 nm) were prepared using a protocol based on the Martin method [60]. Briefly, 5.67 mg of NaBH_4_ in 50 mM NaOH solution (3 mL) and 19.6 mg of HAuCl_4_∙3H_2_O in 50 mM HCl solution (1 mL) were mixed in D.W. (96 mL) while stirring with a magnetic bar. After some time, the color of the mixture changed to ruby red. The reaction was allowed to proceed for 1 h, after which the Au nanoseeds were stored at 4 ℃ for 2–3 days before use.

To fabricate Au nanoseeds introduced SiO_2_ NPs (SiO_2_@Au), 2 mg of aminated SiO_2_ NPs (dispersed in 200 μL ethanol), Au nanoseeds mixture (10 mL), and 20 mg of PVP were mixed. The mixture was incubated overnight and the resulting SiO_2_@Au NPs were washed three times with ethanol by centrifugation at 8,500 rpm for 10 min. The concentration of SiO_2_@Au NPs was adjusted to 1 mg mL^−1^ using 2% (w/v) PVP solution.

### Fabrication of NPs with red, yellow, orange, brown, magenta, purple, and navy colors

To fabricate NPs with multiple colors of PINs, 0.2 mg of SiO_2_@Au (in 200 μL of PVP solution) was mixed with 9.8 mL of PVP solution, and the mixture was stirred at 500 rpm with a magnetic bar. While stirring, Au and/or Ag precursors were added to the mixture with ascorbic acid, depending on the color of the NPs desired for fabrication. For fabricating red NPs, 5 mM HAuCl_4_ solution (10 μL), 5 mM AgNO_3_ solution (20 μL), and 5 mM ascorbic acid solution (60 μL) were sequentially added once every 5 min, repeated six times. For yellow, orange, and brown NPs, 10 mM AgNO_3_ solution (20 μL) and 10 mM ascorbic acid solution (40 μL) were sequentially added once every 5 min, repeated 2, 5, and 11 times, respectively. For magenta, purple, and navy NPs, 10 mM HAuCl_4_ solution (20 μL) and 10 mM ascorbic acid solution (40 μL) were sequentially added once every 5 min, repeated three, seven, and 11 times, respectively. The reaction mixtures were stirred for an additional 10 min after the final addition of precursors. Next, the NPs were washed with ethanol five times via centrifugation at 8,500 rpm for 10 min and redispersed in 0.2 mL of ethanol.

### Conjugation of antibodies onto the fabricated multi-colored PINs

Prior to the conjugation of the antibodies onto the surface of the fabricated PINs, 11-MUA was attached to the surface of the PINs. Briefly, 0.1 mg (in 90 μL of ethanol) of PINs was mixed with 10 μL of 2 mM 11-MUA in ethanol. The mixture was allowed to react at room temperature for 1 h and washed three times with ethanol via centrifugation at 8,500 rpm for 10 min. To activate the carboxylic acid group of 11-MUA, 0.1 mg of washed PINs (in 100 μL of ethanol) were dispersed in 700 μL of 50 mM MES. Subsequently, 100 μL of D.W. containing 2 mM EDC and 100 μL of D.W. containing 2 mM sulfo-NHS were added sequentially and allowed to react at room temperature for 30 min. The supernatant was removed after centrifugation at 10,000 rpm for 10 min. The activated PINs were redispersed in 1 mL of 50 mM MES. Next, 10 μL of antibody solution (1 mg mL^−1^ in PBS) was added to each corresponding PIN and mixed. The mixture was allowed to react at room temperature for 2 h, followed by washing with 1 mL of 50 mM MES via centrifugation at 10,000 rpm for 10 min. To deactivate the carboxyl group remaining activated in the antibody-conjugated PINs, 3.2 μL of ethanolamine was added and reacted at room temperature for 30 min. After completion of the reaction, the PINs were washed twice with 50 mM MES via centrifugation at 10,000 rpm for 10 min. Subsequently, the PINs were sequentially washed with 0.5% (v/v) PBST and 0.5% (w/v) BSA/PBS via centrifugation at 10,000 rpm for 10 min. The washed PINs were redispersed in 1 mL of 0.5% (w/v) BSA/PBS and stored in a refrigerator at 4 °C. For the CLFA experiment using a strip, the solvent in which the probe was dispersed was changed to 1 mL of 0.5% (v/v) PBST to maintain a consistent developing solution.

### Preparation of test strips for the multi-colored PIN-based CLFA

The test strips for the CLFA were prepared according to a previously published method with minor modifications [49]. After assembling the NC membrane on the backing card, 1 mg mL^−1^ of goat anti-mouse IgG Ab solution was dispensed onto the control line. Subsequently, for PSA detection, an anti-PSA antibody (14801) solution (1 mg mL^−1^ in PBS) was dispensed as a test line. For multiplex detection, 1 mg mL^−1^ of ICAM1 capture antibody (1 mg mL^−1^ in PBS, for T_1_), CA19-9 monoclonal antibody (A46300) solution (1 mg mL^−1^ in PBS, for T_2_), and anti-PSA antibody (14801) solution (1 mg mL^−1^ in PBS, for T_3_) were dispensed. The distance between the control line and each test line was maintained at a minimum of 4.0 mm, and the distance between each test line was maintained at a minimum of 2.0 mm. All solutions were dispensed at a flow rate of 0.6 μL cm^−1^. The antibody-dispensed NC membrane was dried in a desiccator for approximately 2 h, and the absorbent pad was attached to the backing card and cut to a width of 4 mm to complete the CLFA kit.

### Detection of biomarkers of prostate cancer in samples by using the PIN-based CLFA

To confirm that the specific biomarker detection abilities of the various colored probes were consistent, PSA detection was performed using the brown, navy, and red PINs. First, 1 mg mL^−1^ goat anti-mouse IgG Ab solution was dispensed onto the control line, and 1 mg mL^−1^ anti-PSA antibody (14801) solution was dispensed onto the test line. Next, Brown-PSA Ab, Navy-PSA Ab, and Red-PSA Ab mixtures (0.1 mg mL^−1^, in 0.5% [v/v] PBST) were synthesized by conjugating three colored particles (brown, navy, and red PINs) with anti-PSA antibody (14803). Subsequently, PSA solutions were prepared with concentrations of 0, 0.004, 0.04, 0.4, 4, 40, 400, and 4000 ng mL^−1^ (10 μL in PBS). For the assay, 3 μL of each PSA solution was added to eight wells of a 96-well plate, followed by 3 μL of Brown-PSA Ab mixture (0.1 mg mL^−1^, in 0.5% [v/v] PBST) and 27 μL of 0.5% (v/v) PBST. The strips with 1 mg mL^−1^ of anti-PSA antibody dispensed on the test line were then immersed in each well for single detection. Single detection using Navy-PSA Ab and Red-PSA Ab mixtures was performed in the same manner as described above.

### Multiplex analysis using the multi-colored PIN-based CLFA

To confirm the possibility of quantitative multiplexing using biomarkers of specific diseases, ICAM1, CA19-9, and PSA, which are biomarkers for gastric, pancreatic, and prostate cancers, respectively, were used as targets. Brown, navy, and red PINs were selected as the probes. Brown-ICAM1 antibody mixture (0.1 mg mL^−1^, in 0.5% [w/v] BSA/PBS) was prepared by conjugating ICAM1 Capture Ab (ab288941) to brown PIN. Similarly, navy-CA19-9 (0.1 mg mL^−1^, in 0.5% [w/v] BSA/PBS) and red-PSA antibody mixtures (0.1 mg mL^−1^, in 0.5% [w/v] BSA/PBS) were prepared by conjugating CA19-9 monoclonal antibody (A46400) and anti-PSA antibody (14803) to navy and red PINs, respectively. As next step, antigen sample solutions were prepared as follows. To prepare antigen solution with the highest concentration, 4 μL of ICAM1 solution (40,000 ng mL^−1^ in PBS), CA19-9 solution (37,000 U mL^−1^ in PBS), and PSA solution (40,000 ng mL^−1^ in PBS) were mixed, and additional seven kind of antigen solutions were prepared with tenfold serial dilution. After preparation of sample solution, 3 μL of brown-ICAM1-Ab (0.1 mg mL^−1^, in 0.5% PBST), navy-CA19-9-Ab (0.1 mg mL^−1^, in 0.5% PBST), red-PSA-Ab (0.1 mg mL^−1^, in 0.5% PBST), and 21 μL of 0.5% PBST were put into individual well of 96-well plate and mixed. Subsequently, the 9 μL of antigen solutions with different concentration include negative control were put into each well, and then, prepared test strips for multiplex analysis were immersed into each well.

## Supplementary Information

Supplementary material 1. The following files are available free of charge. Low magnification TEM images of fabricated NPs; Photograph images of fabricated PINs after storage; TEM images of fabricated PINs after storage; TEM images of fabricated PINs with five different batches; Calculation of limit of detectionfor each biomarker in the CLFA system

## Data Availability

All data generated or analyzed during this study are included in this manuscript and its supplementary material.

## Abbreviations

LFA: Lateral flow assay
AuNPs: Gold nanoparticles
PINs: Plasmon-controlled metal–silica Isoform Nanocomposites
LOD: Limit of detection
POCT: Point-of-care test
CLFAs: Colorimetric lateral flow assays
NPs: Nanoparticles
TEM: Transmission electron microscopy
EDS: Energy-dispersive spectroscopy
NC: Nitrocellulose
PSA: Prostate-specific antigen
11-MUA: 11-Mercaptoundecanoic acid
ICAM1: Intercellular adhesion molecule 1
CA19-9: Carbohydrate antigen 19-9
TEOS: Tetraethylorthosilicate
APTES: 3-Aminopropyltriethoxysilane
HAuCl_4_∙3H_2_O: Gold (III) chloride trihydrate
AgNO_3_: Silver nitrate
EDC: 1-Ethyl-3-(3-dimethylaminopropyl)carbodiimide hydrochloride
sulfo-NHS: N-hydroxysulfosuccinimide
NaBH_4_: Sodium borohydride
MES: 4-Morpholineethanesulfonic acid
PVP: Poly(vinylpyrrolidone)
KOH: Potassium hydroxide
NaOH: Sodium hydroxide
HCl: Hydrochloric acid
PBS: Phosphate-buffered saline
PBST: Tween 20 in phosphate-buffered saline
D.W.: Deionized water
