# Supplementary material for: Highly sensitive multiplexed colorimetric lateral flow immunoassay by plasmon-controlled metal–silica isoform nanocomposites: PINs
Source: Nano Converg. 2024 Oct 24;11:42. doi: 10.1186/s40580-024-00449-y (PMC11502615; doi:10.1186/s40580-024-00449-y)
Supplement: Supplementary file 1 — Supplementary material 1. The following files are available free of charge. Low magnification TEM images of fabricated NPs; Photograph images of fabricated PINs after storage; TEM images of fabricated PINs after storage; TEM images of fabricated PINs with five different batches; Calculation of limit of detectionfor each biomarker in the CLFA system [file 40580_2024_449_MOESM1_ESM.docx]

**[Supporting Information]**

**Highly Sensitive Multiplexed Colorimetric Lateral Flow Immunoassay by Plasmon-controlled metal-silica Isoform Nanocomposites: PINs**

Minsup Shin,^1†^ Wooyeon Kim,^1†^ Kwanghee Yoo,^1^ Hye-Seong Cho,^1^ Sohyeon Jang,^1^ Han-Joo Bae,^1^ Jaehyun An,^2^ Jong-chan Lee,^3^ Hyejin Chang,^4^ Dong-Eun Kim,^1^ Jaehi Kim,^1*^ Luke P. Lee,^5,6,7*^ and Bong-Hyun Jun^1*^

Figure S1. Low magnification TEM images of (a) SiO_2_@Au, (b) red-, (c) yellow-, (d) orange-, (e) brown-, (f) magenta-, (g) purple-, and (h) navy-colored PINs. The scale bar represents 200 nm.

Figure S2. Photograph images of fabricated PINs after storage.


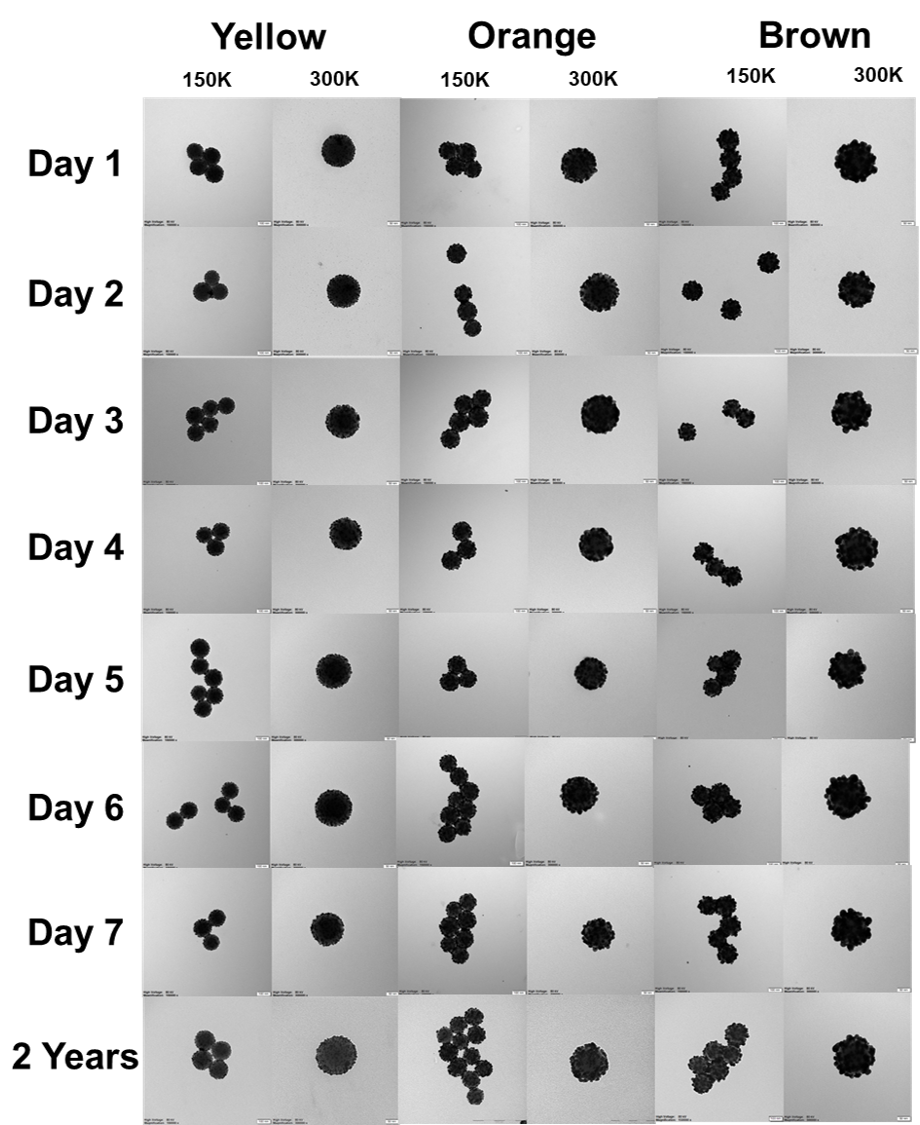


Figure S3. TEM images of fabricated PINs after storage.


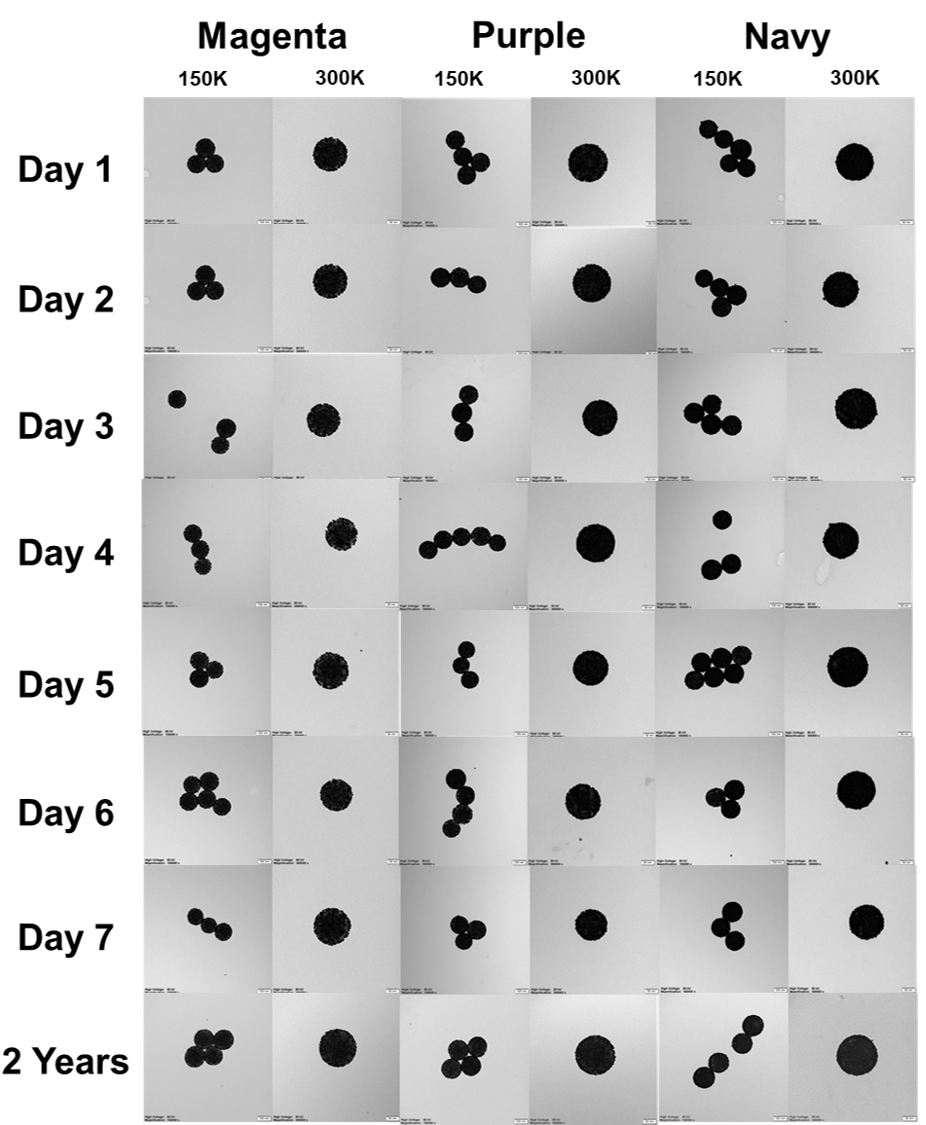


Figure S3. *(cont’d)*.


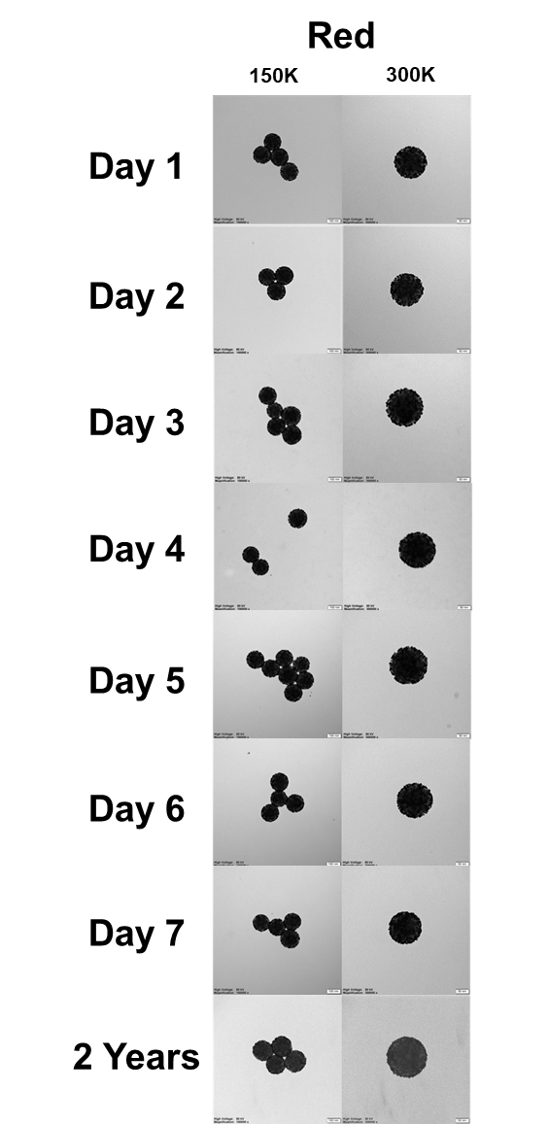


Figure S3. *(cont’d)*.


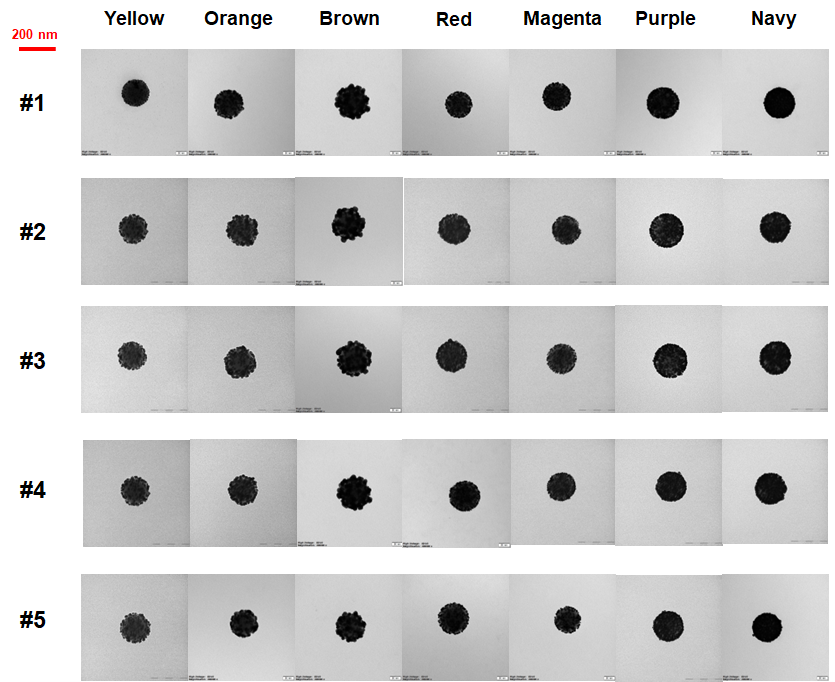


**Figure S4.** TEM images of fabricated PINs with five different batches.

**Supplementary Note: Calculation of** **limit of detection (LOD) for each biomarker in the CLFA system**

Quantitative analysis of CLFA using the ImageJ program and fitting the results to a sigmoidal curve in the Origin program **(Figure S5)**[1]:

1. Run the strip image file in the ImageJ program.
2. Select the straight command option, set the line to include the test line drawn on the NC membrane, and extract the value through the plot profile.
3. The signal value of the test line is obtained by subtracting the background signal from the peak.
4. Input the obtained signal value into Origin and fit it into a logistic (sigmoidal) curve.
5. Then, the LOD was calculated by substituting the variables into the fitted curve equation (The quantitative curve for each experimental result was obtained by analyzing three independent CLFAs (n = 3)).

**Figure S5.** A) Run the ImageJ program and select the straight-line command. B) Select the width of the straight command to measure signal values. C) Measure the signal value of the actual strip with the straight command. D) Control, test, and background peaks are shown in the plot profile.

**Table S2.** LOD calculation parameters for each biomarker

The fitted curve corresponds to the following equation: $y=START+\left( END-START \right)\times x^{n}/(k^{n}+x^{n})$

(The y value is obtained by multiplying the standard deviation of the signal value corresponding to the lowest concentration of each fitted curve by 3.3.)

|  | **Single detection** | | | **Multiple detection** | | |
| --- | --- | --- | --- | --- | --- | --- |
| **Parameters** | **PSA**  **(Brown NP)** | **PSA**  **(Navy NP)** | **PSA**  **(Red NP)** | **ICAM1**  **(Brown NP)** | **CA19-9**  **(Navy NP)** | **PSA**  **(Red NP)** |
| **START** | 330.83 | 591.44 | 258.96 | 22.60 | 2.54 | -0.36 |
| **END** | 7191.42 | 6435.34 | 6009.90 | 138.81 | 99.75 | 94.54 |
| **k**  **(Michaelis constant)** | 17.46 | 68.63 | 130.53 | 679.90 | 65.50 | 106.27 |
| **n**  **(Coefficient)** | 1.95 | 0.91 | 0.72 | 1.12 | 0.46 | 0.54 |
| **R-square** | 0.99 | 0.99 | 0.99 | 0.99 | 0.99 | 0.99 |

1. Parolo C, Sena-Torralba A, Bergua JF, Calucho E, Fuentes-Chust C, Hu L, Rivas L, Álvarez-Diduk R, Nguyen EP, Cinti S: **Tutorial: design and fabrication of nanoparticle-based lateral-flow immunoassays.** *Nature protocols* 2020, **15:**3788-3816.
